# Supplementary material for: Anti-Inflammatory Responses Produced with Nippostrongylus brasiliensis-Derived Uridine via the Mitochondrial ATP-Sensitive Potassium Channel and Its Anti-Atherosclerosis Effect in an Apolipoprotein E Gene Knockout Mouse Model
Source: Biomolecules. 2024 Jun 8;14(6):672. doi: 10.3390/biom14060672 (PMC11201709; doi:10.3390/biom14060672)
Supplement: Supplementary file 1 [file biomolecules-14-00672-s001.zip › biomolecules-3021225-Supplementary information S2.pdf]

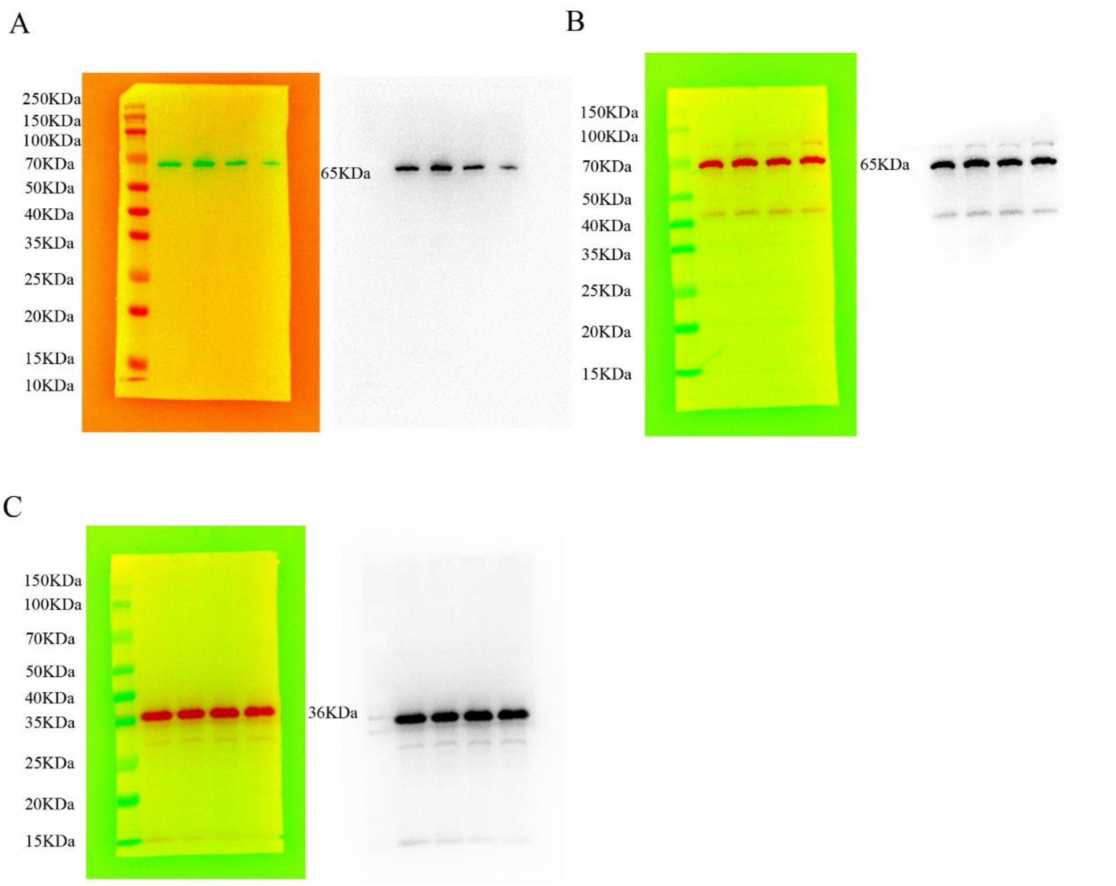

Supplementary information S2. (A) The full length gels and blots from detection of phosphorylation levels of NF-κB. **(B)** The full length gels and blots from detection of NF-κB levels. **(C)** The full length gels and blots from detection of GAPDH levels.
