# Supplementary figures and images for: Anti-Inflammatory Responses Produced with Nippostrongylus brasiliensis-Derived Uridine via the Mitochondrial ATP-Sensitive Potassium Channel and Its Anti-Atherosclerosis Effect in an Apolipoprotein E Gene Knockout Mouse Model
Source: Biomolecules. 2024 Jun 8;14(6):672. doi: 10.3390/biom14060672 (PMC11201709; doi:10.3390/biom14060672)

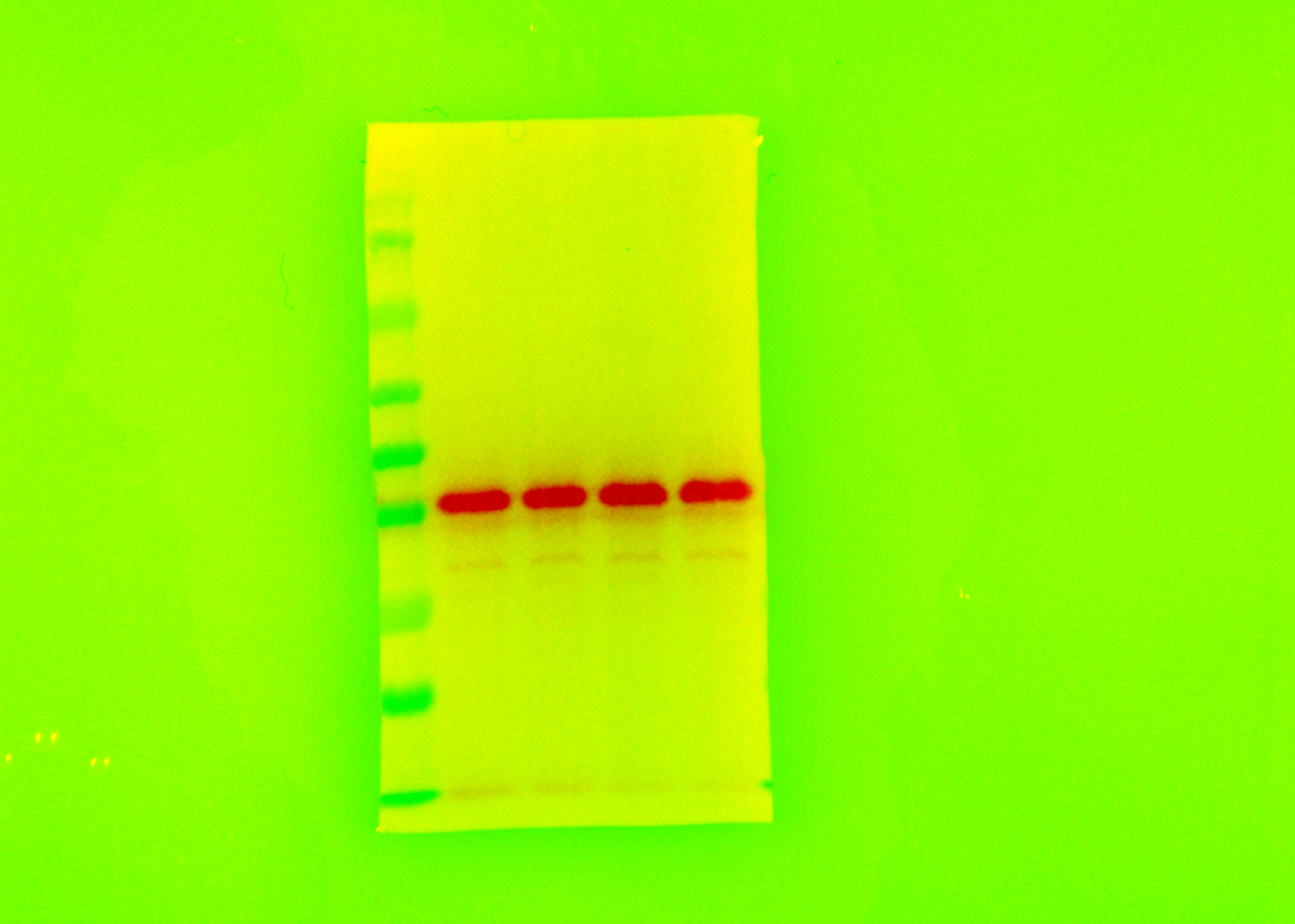

Supplement: Supplementary file 1 [file biomolecules-14-00672-s001.zip › Supplementary information S2 (original images of WB)/GAPDHó┘ -1.tif]

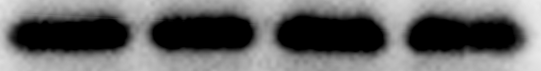

Supplement: Supplementary file 1 [file biomolecules-14-00672-s001.zip › Supplementary information S2 (original images of WB)/GAPDHó┘ screenshot.tif]

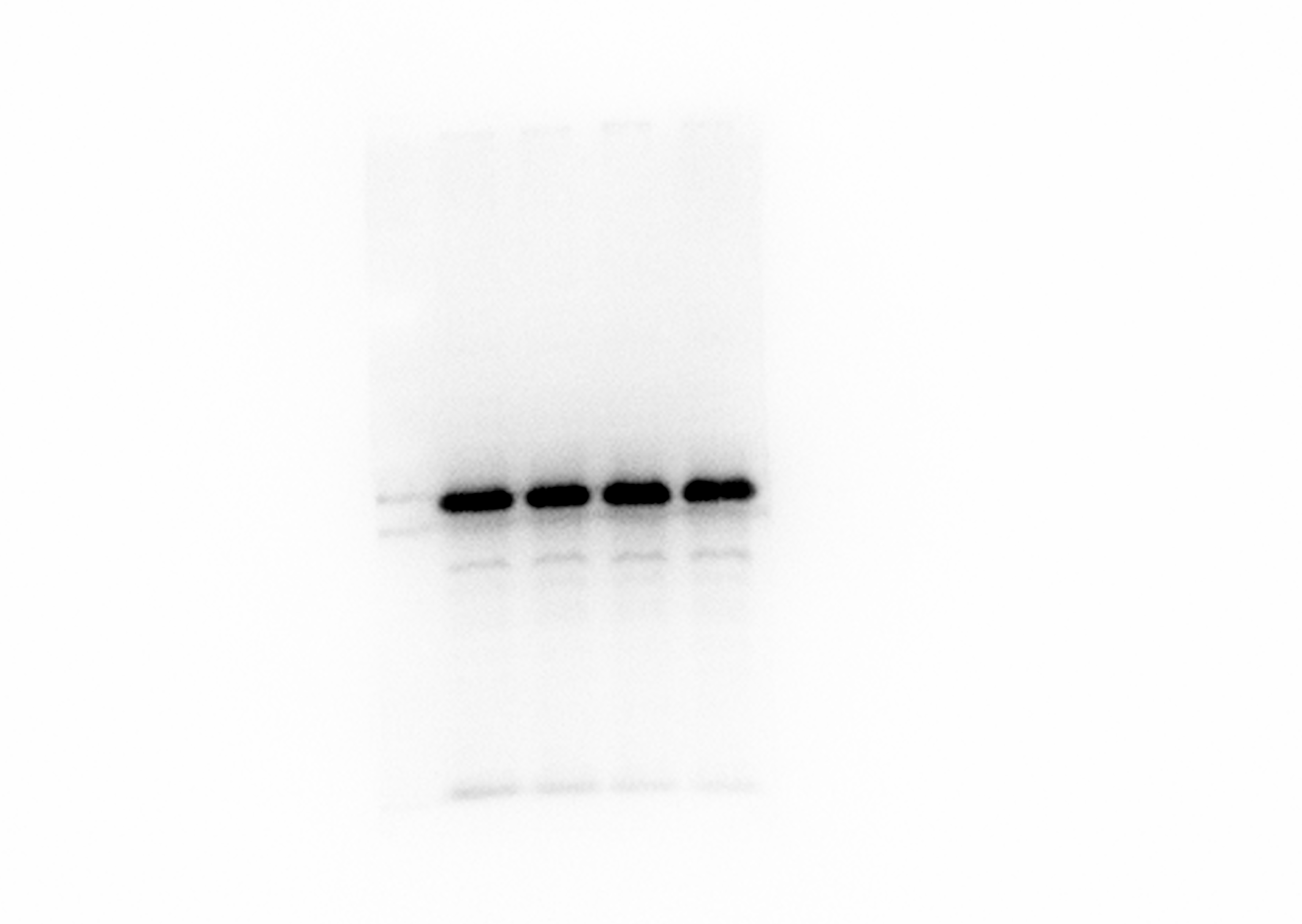

Supplement: Supplementary file 1 [file biomolecules-14-00672-s001.zip › Supplementary information S2 (original images of WB)/GAPDHó┘.tif]

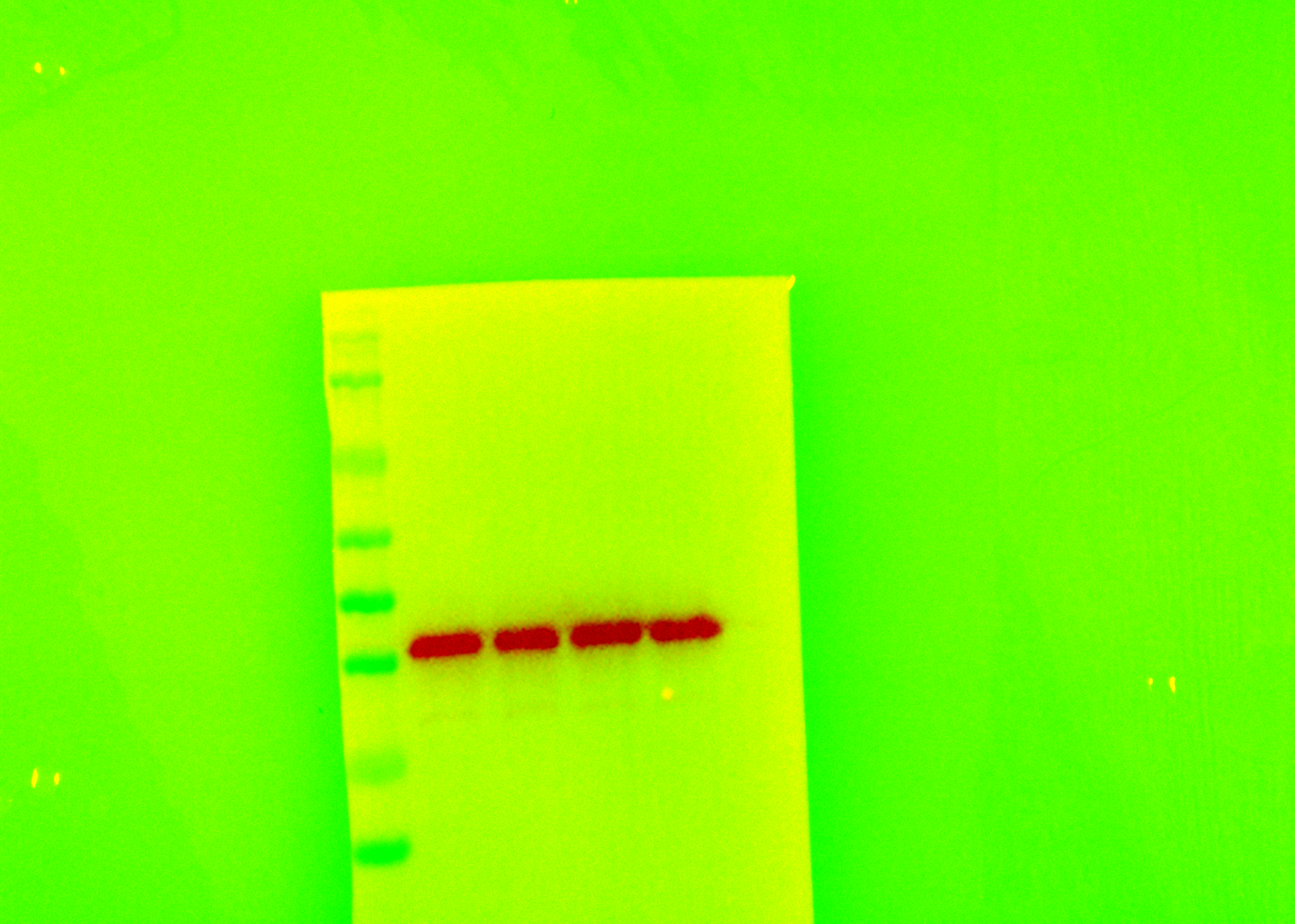

Supplement: Supplementary file 1 [file biomolecules-14-00672-s001.zip › Supplementary information S2 (original images of WB)/GAPDHó┌ -1.tif]

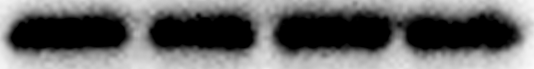

Supplement: Supplementary file 1 [file biomolecules-14-00672-s001.zip › Supplementary information S2 (original images of WB)/GAPDHó┌ screenshot.tif]

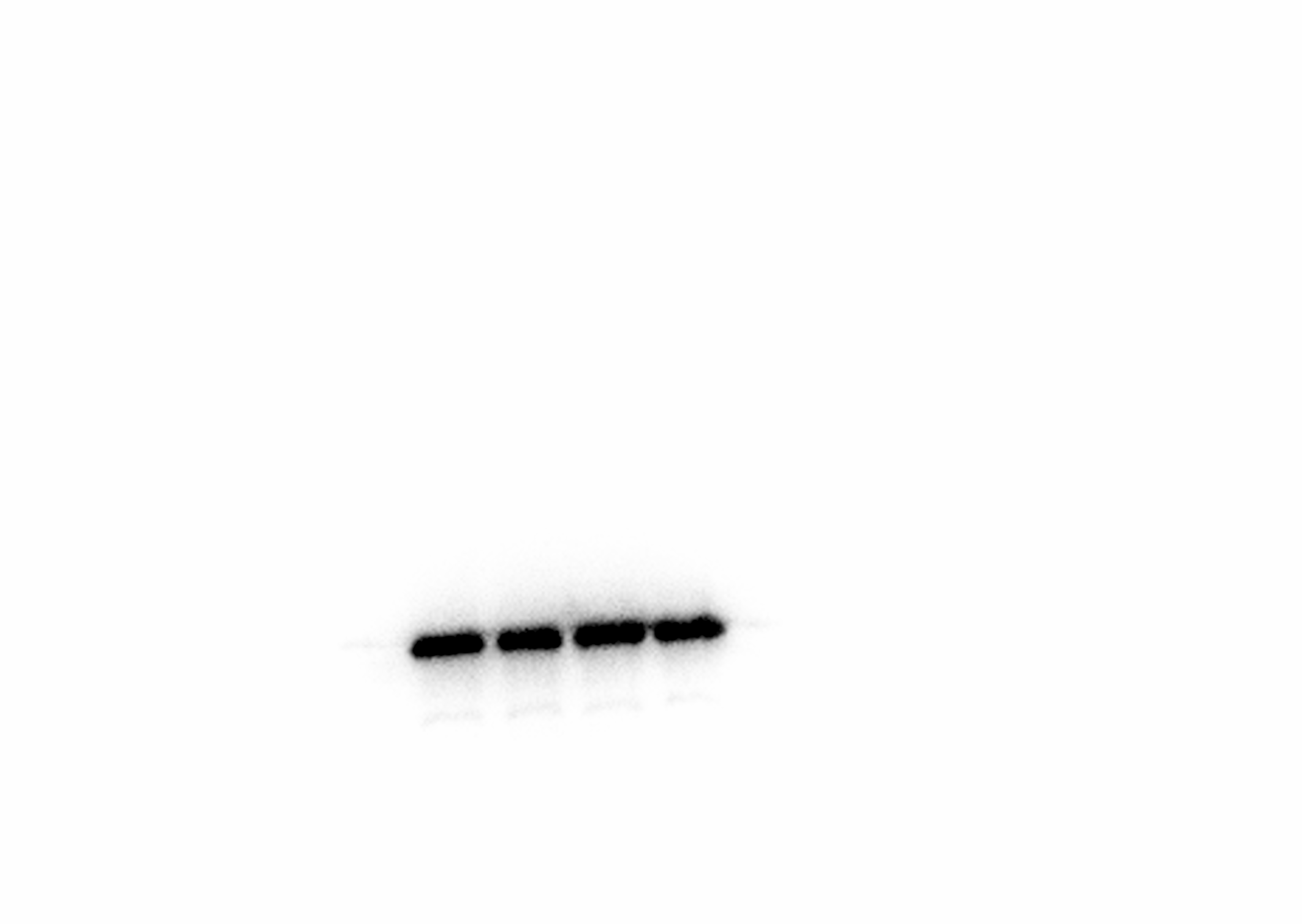

Supplement: Supplementary file 1 [file biomolecules-14-00672-s001.zip › Supplementary information S2 (original images of WB)/GAPDHó┌.tif]

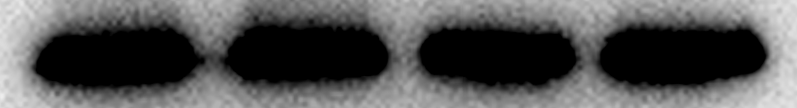

Supplement: Supplementary file 1 [file biomolecules-14-00672-s001.zip › Supplementary information S2 (original images of WB)/GAPDHó█ screenshot.tif]

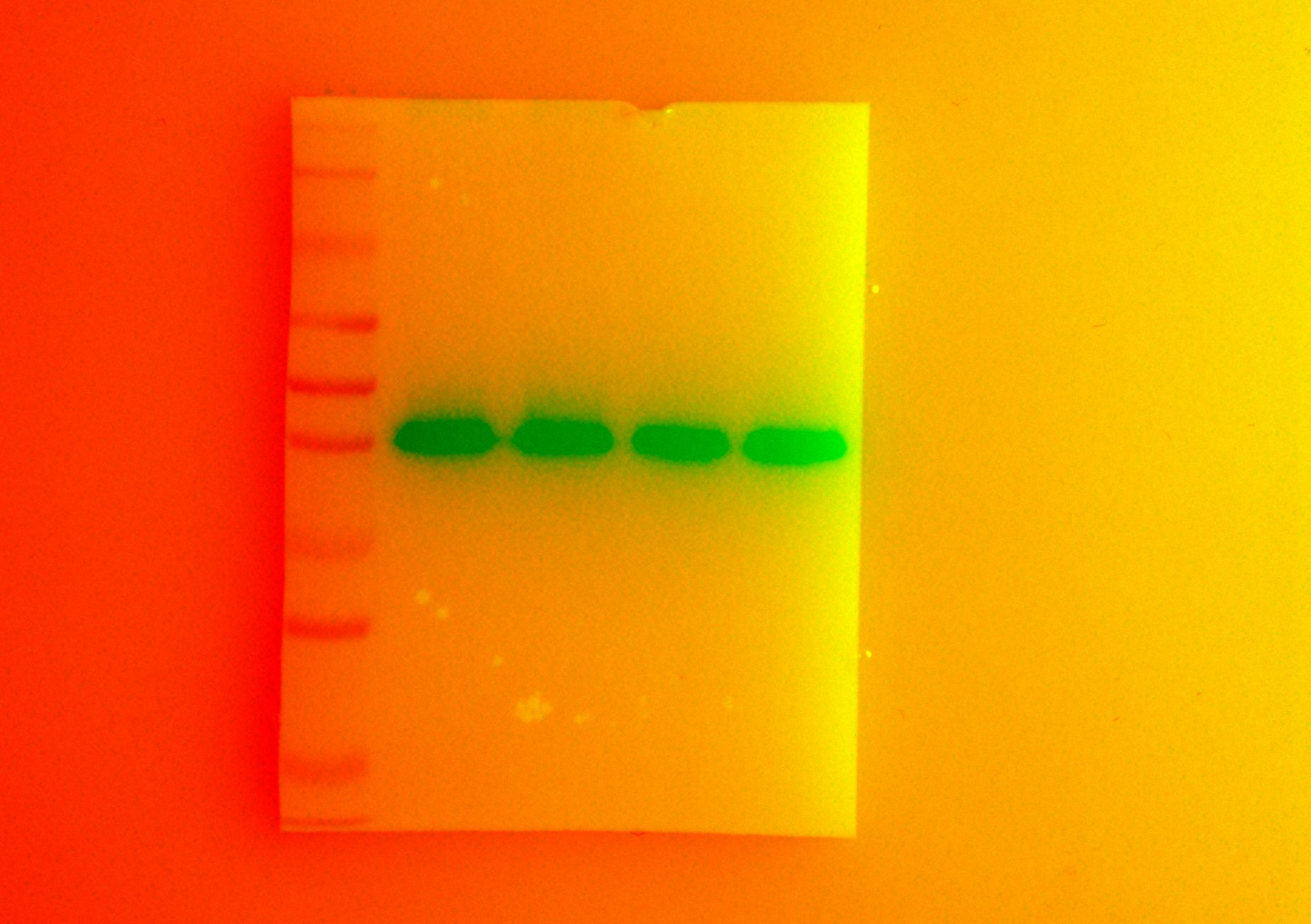

Supplement: Supplementary file 1 [file biomolecules-14-00672-s001.zip › Supplementary information S2 (original images of WB)/GAPDHó█-1.tif]

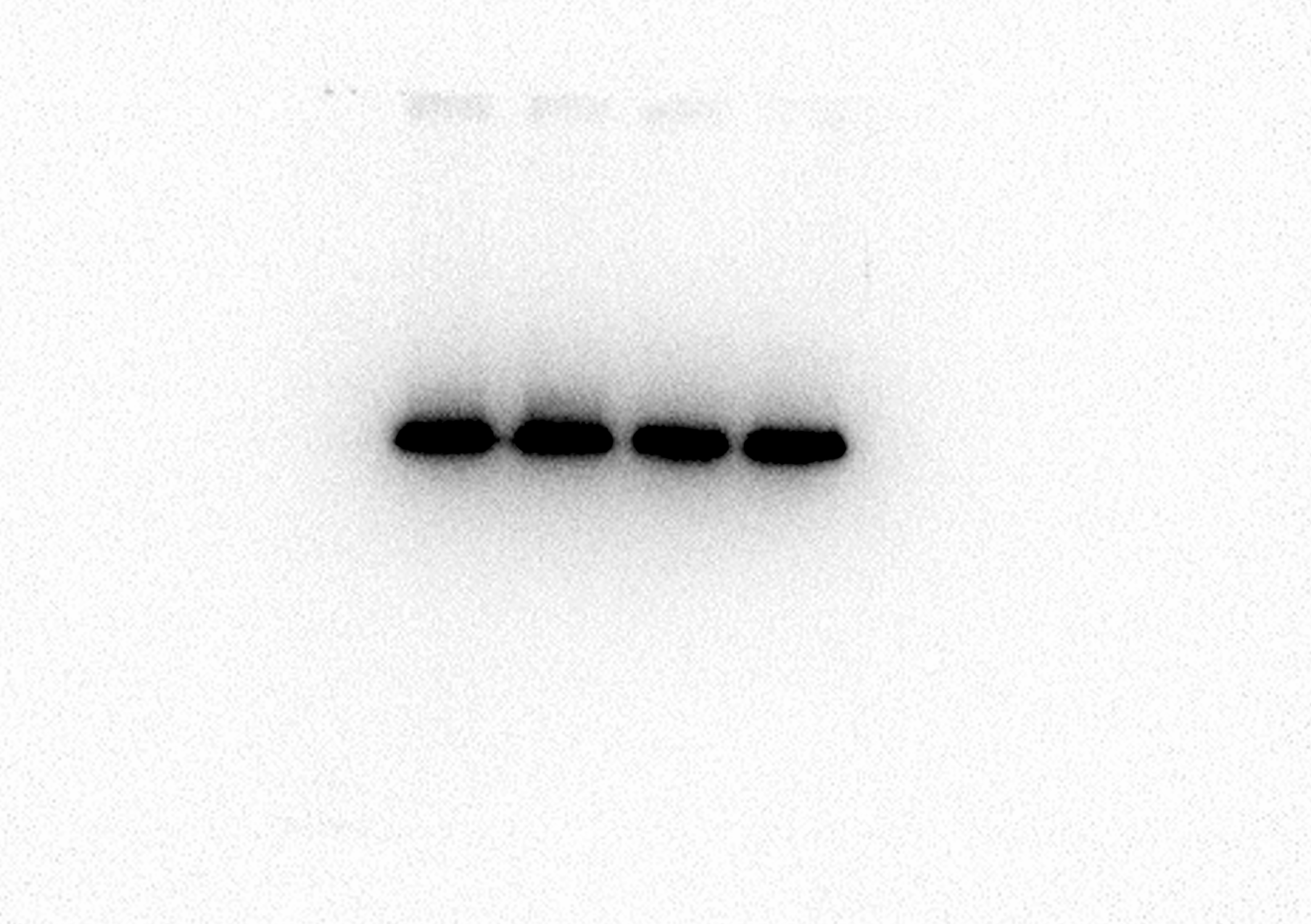

Supplement: Supplementary file 1 [file biomolecules-14-00672-s001.zip › Supplementary information S2 (original images of WB)/GAPDHó█.tif]

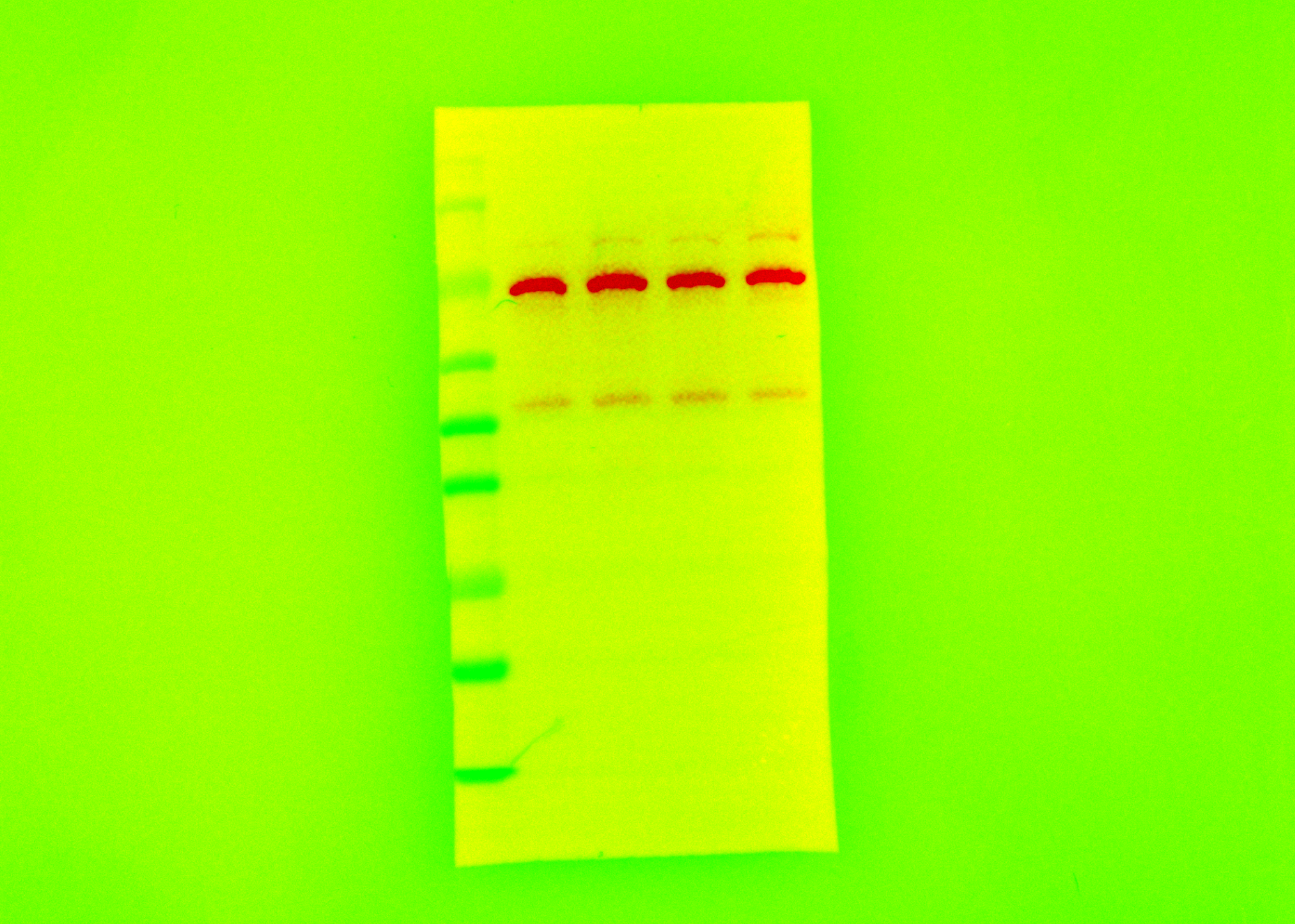

Supplement: Supplementary file 1 [file biomolecules-14-00672-s001.zip › Supplementary information S2 (original images of WB)/NF-kBó┘ -1.tif]

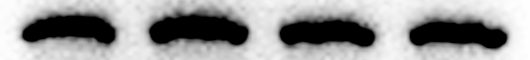

Supplement: Supplementary file 1 [file biomolecules-14-00672-s001.zip › Supplementary information S2 (original images of WB)/NF-kBó┘ screenshot.tif]

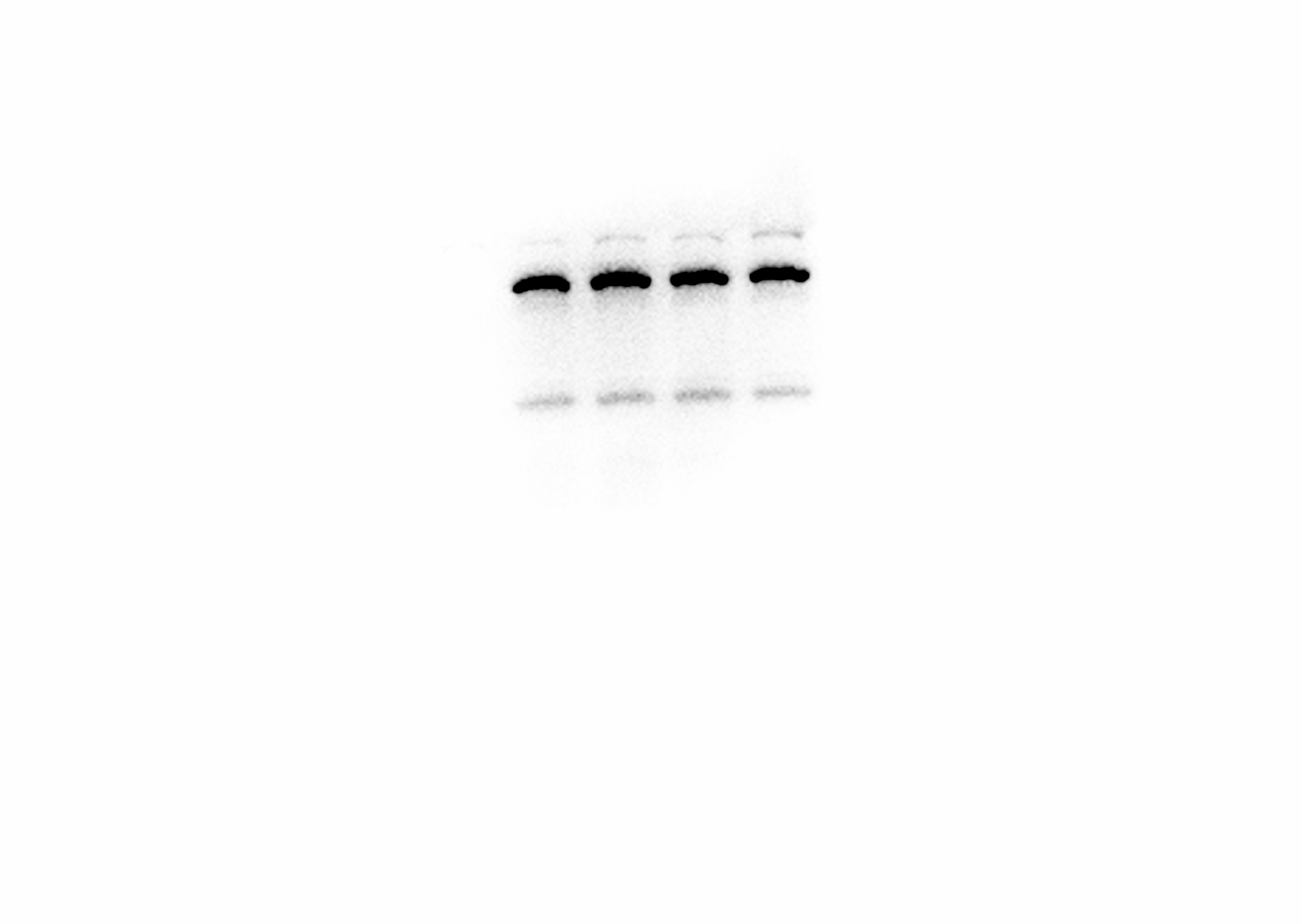

Supplement: Supplementary file 1 [file biomolecules-14-00672-s001.zip › Supplementary information S2 (original images of WB)/NF-kBó┘.tif]

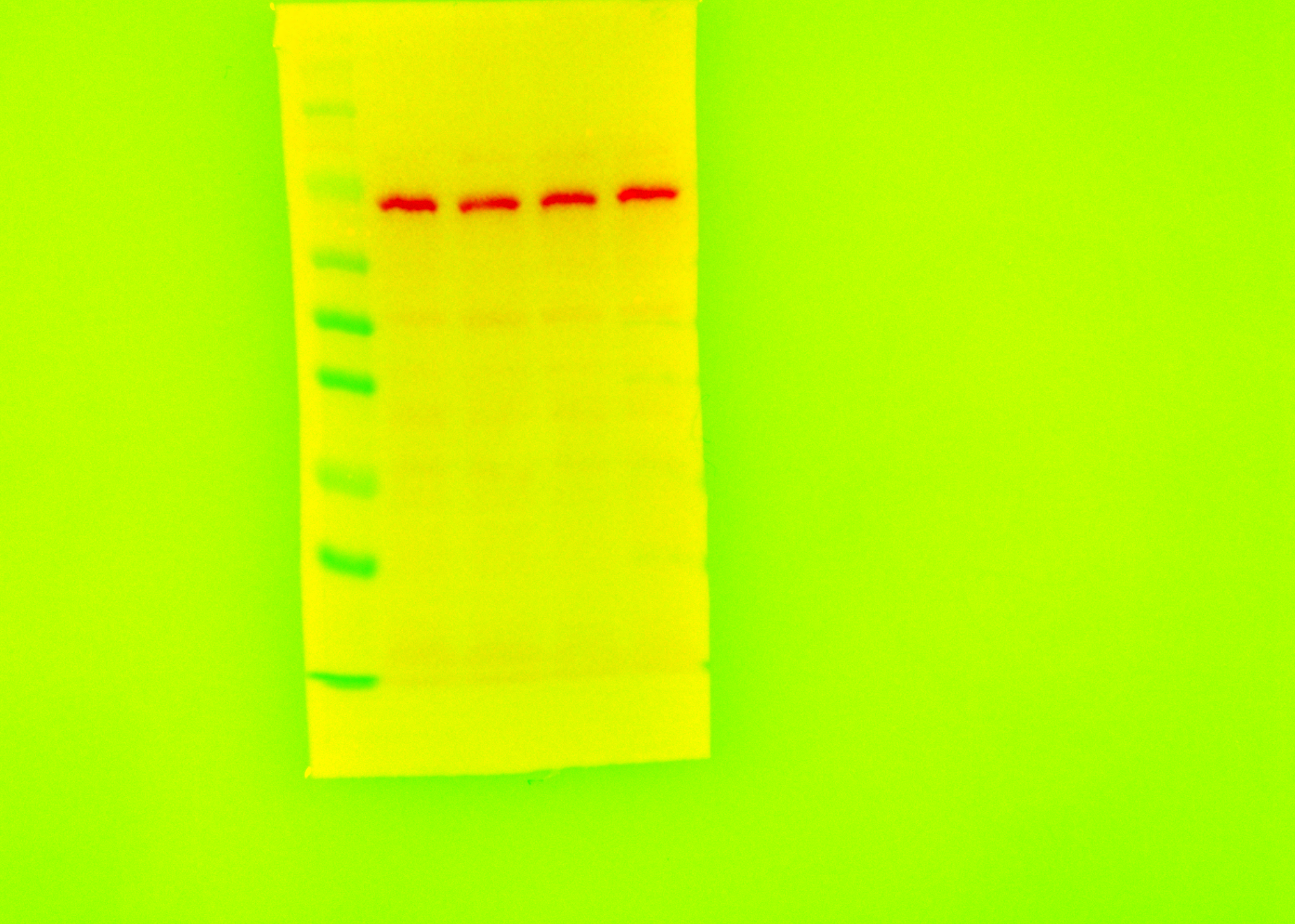

Supplement: Supplementary file 1 [file biomolecules-14-00672-s001.zip › Supplementary information S2 (original images of WB)/NF-kBó┌ -1.tif]

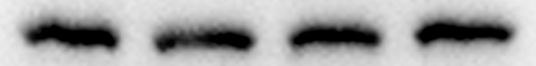

Supplement: Supplementary file 1 [file biomolecules-14-00672-s001.zip › Supplementary information S2 (original images of WB)/NF-kBó┌ screenshot.tif]

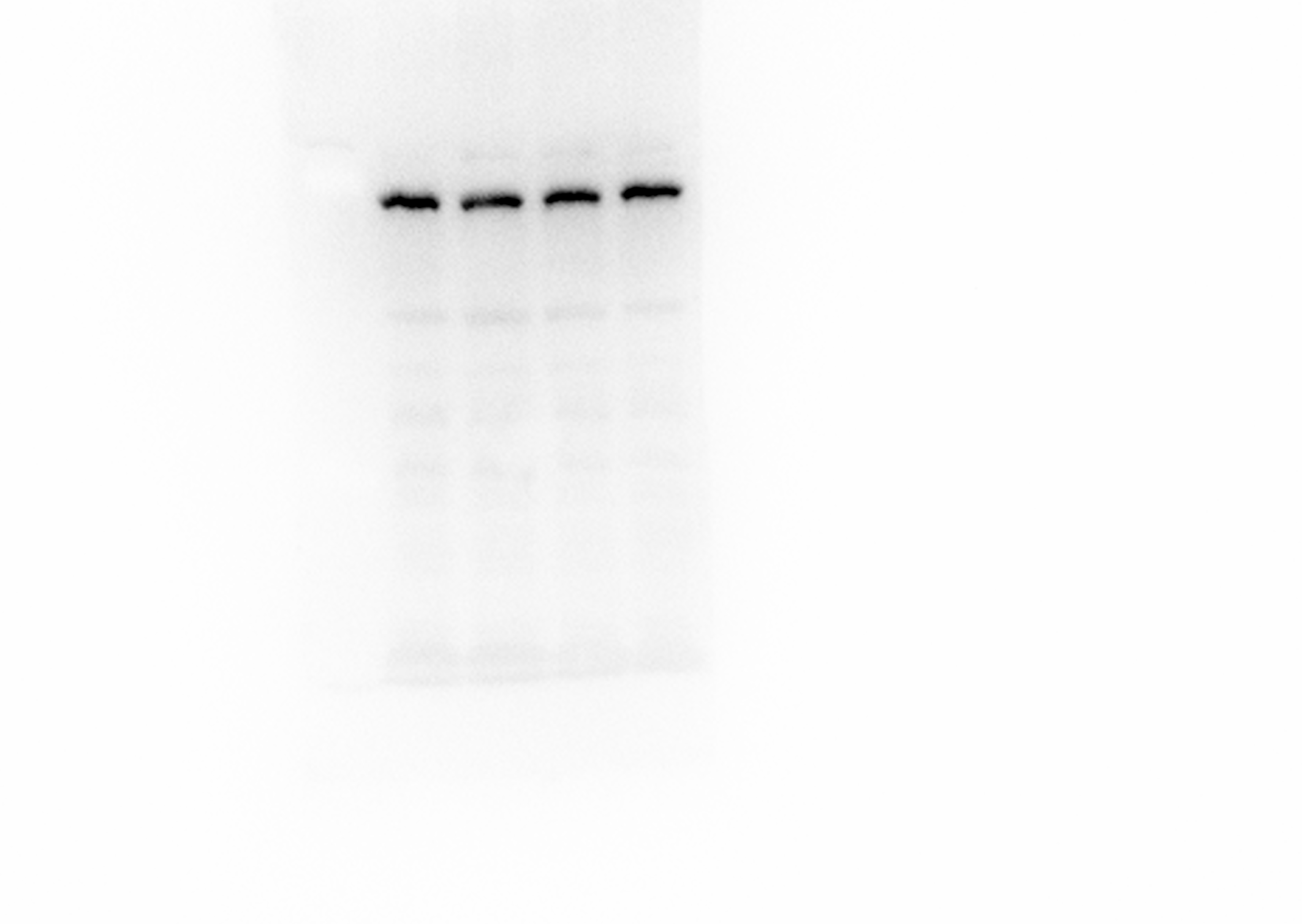

Supplement: Supplementary file 1 [file biomolecules-14-00672-s001.zip › Supplementary information S2 (original images of WB)/NF-kBó┌.tif]

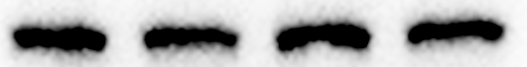

Supplement: Supplementary file 1 [file biomolecules-14-00672-s001.zip › Supplementary information S2 (original images of WB)/NF-kBó█ screenshot.tif]

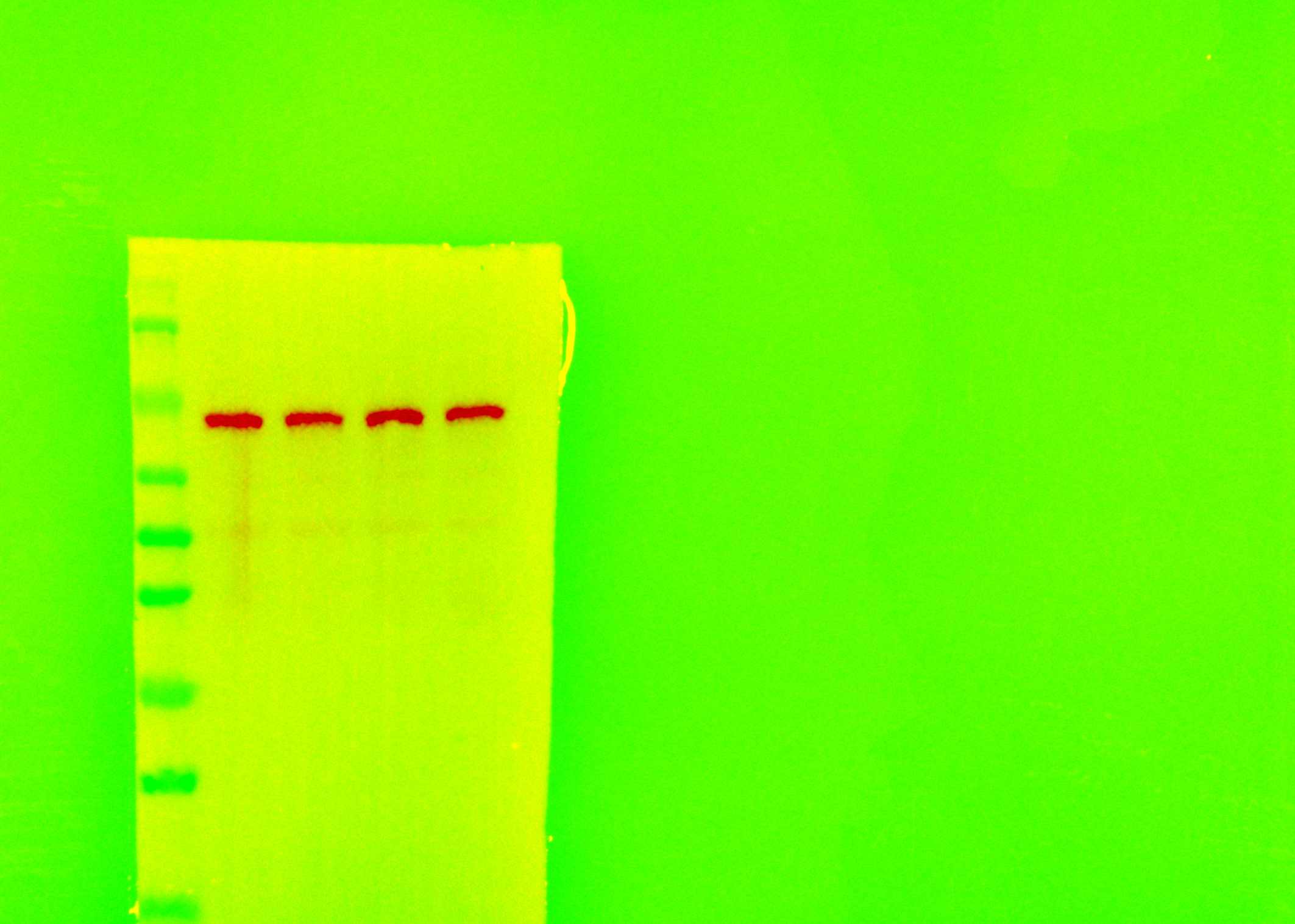

Supplement: Supplementary file 1 [file biomolecules-14-00672-s001.zip › Supplementary information S2 (original images of WB)/NF-kBó█-1.tif]

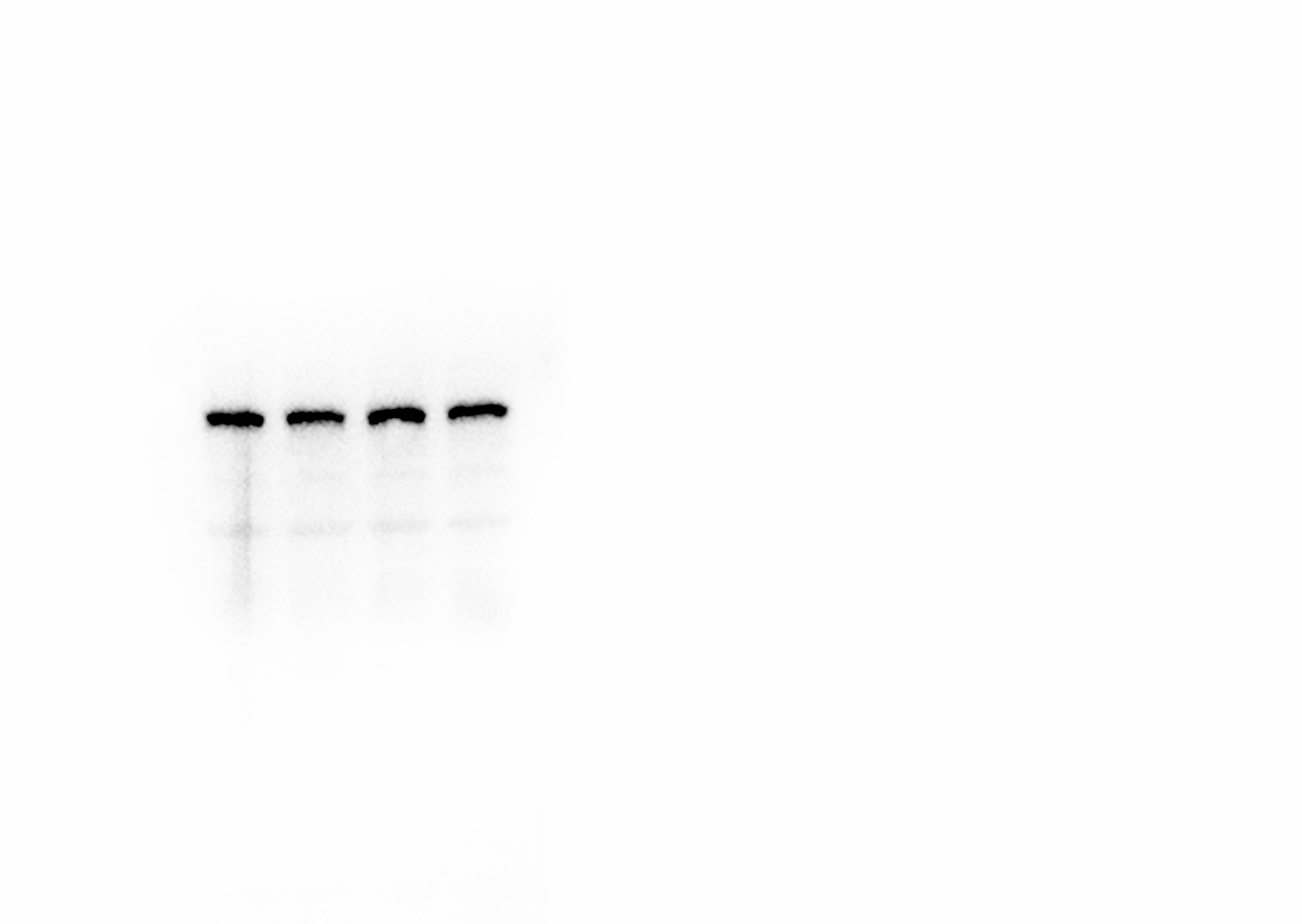

Supplement: Supplementary file 1 [file biomolecules-14-00672-s001.zip › Supplementary information S2 (original images of WB)/NF-kBó█.tif]

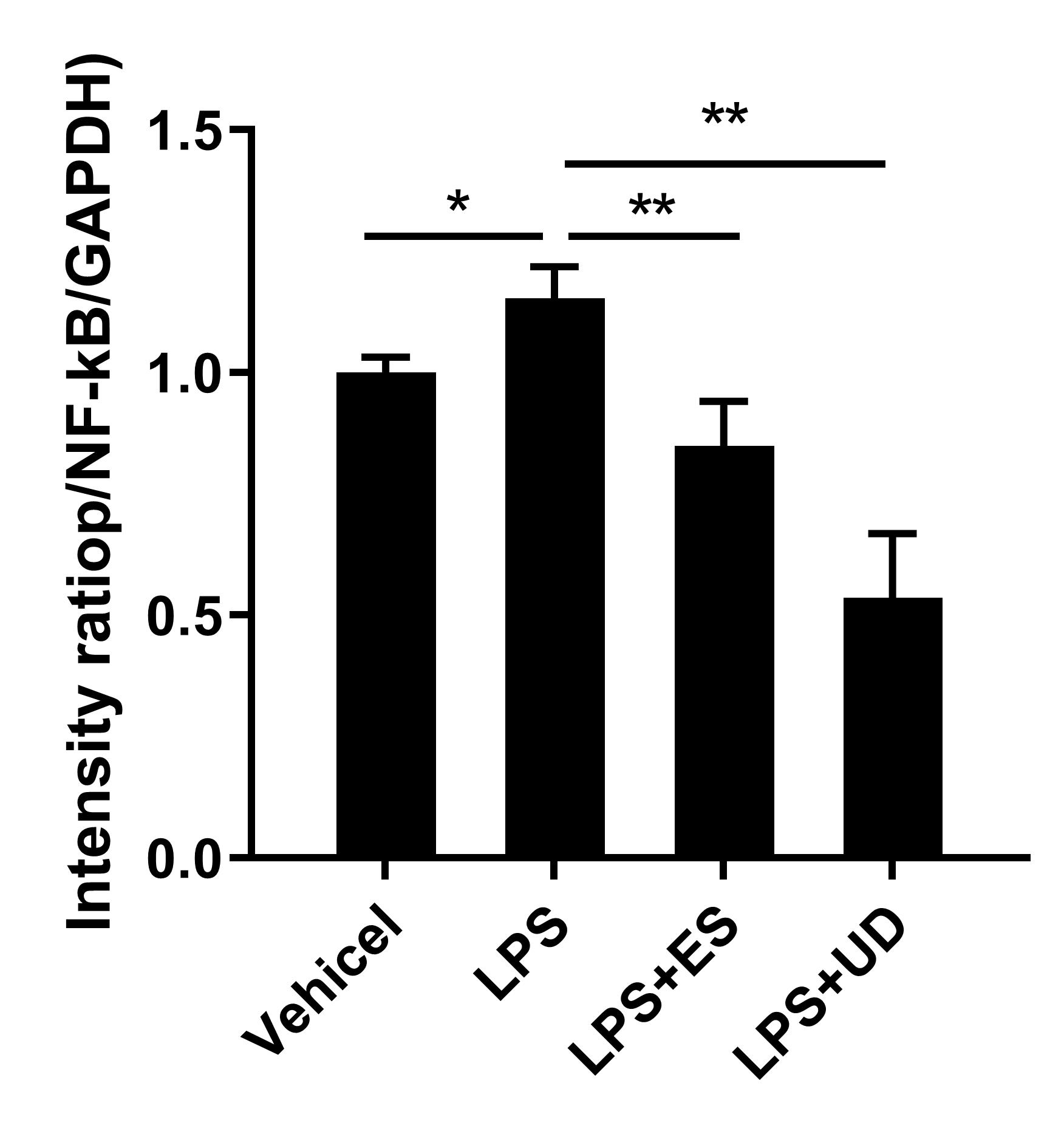

Supplement: Supplementary file 1 [file biomolecules-14-00672-s001.zip › Supplementary information S2 (original images of WB)/NF-a╩B.jpg]

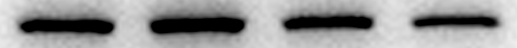

Supplement: Supplementary file 1 [file biomolecules-14-00672-s001.zip › Supplementary information S2 (original images of WB)/p-NF-kBó┘ screenshot.tif]

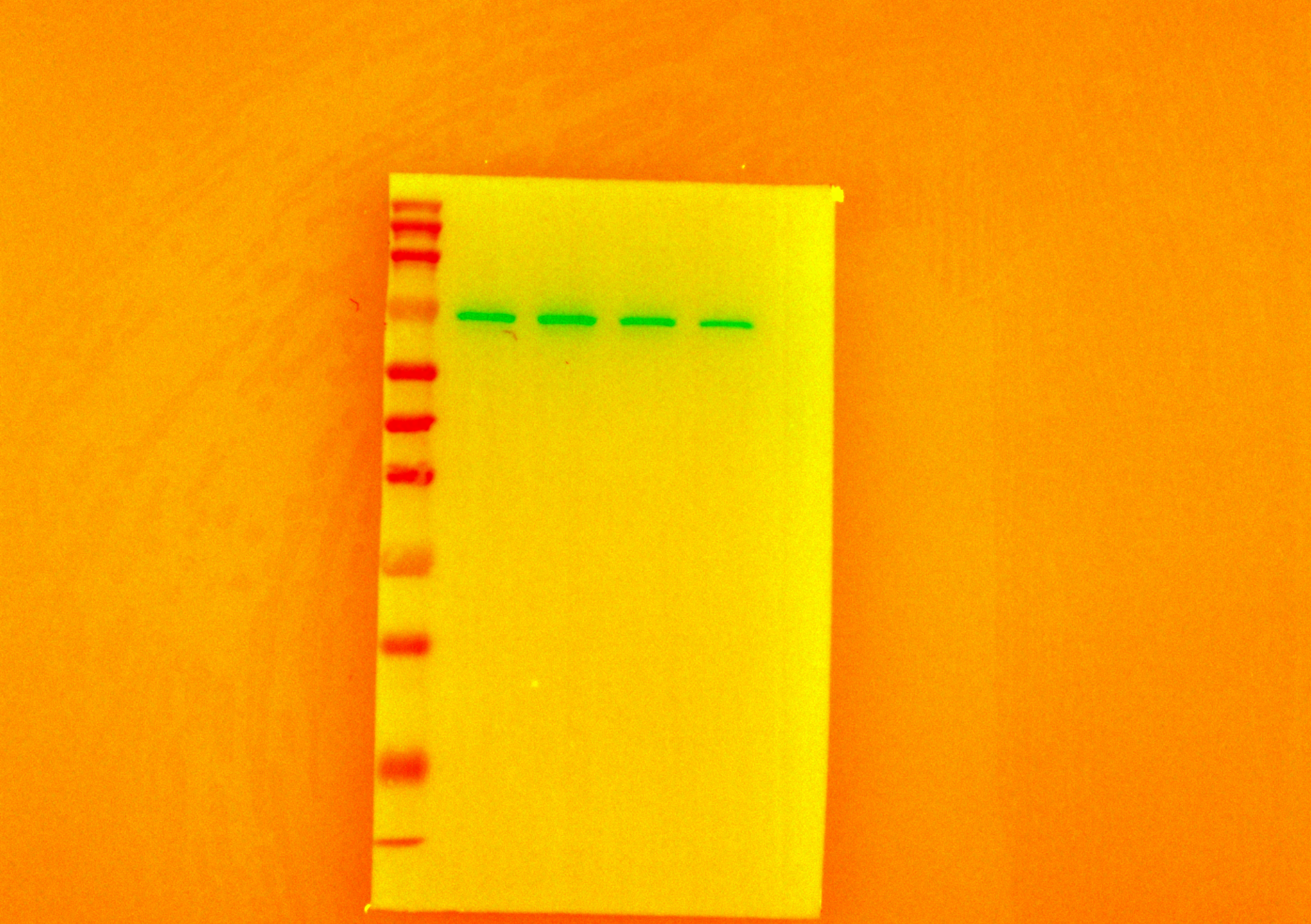

Supplement: Supplementary file 1 [file biomolecules-14-00672-s001.zip › Supplementary information S2 (original images of WB)/p-NF-kBó┘-1.tif]

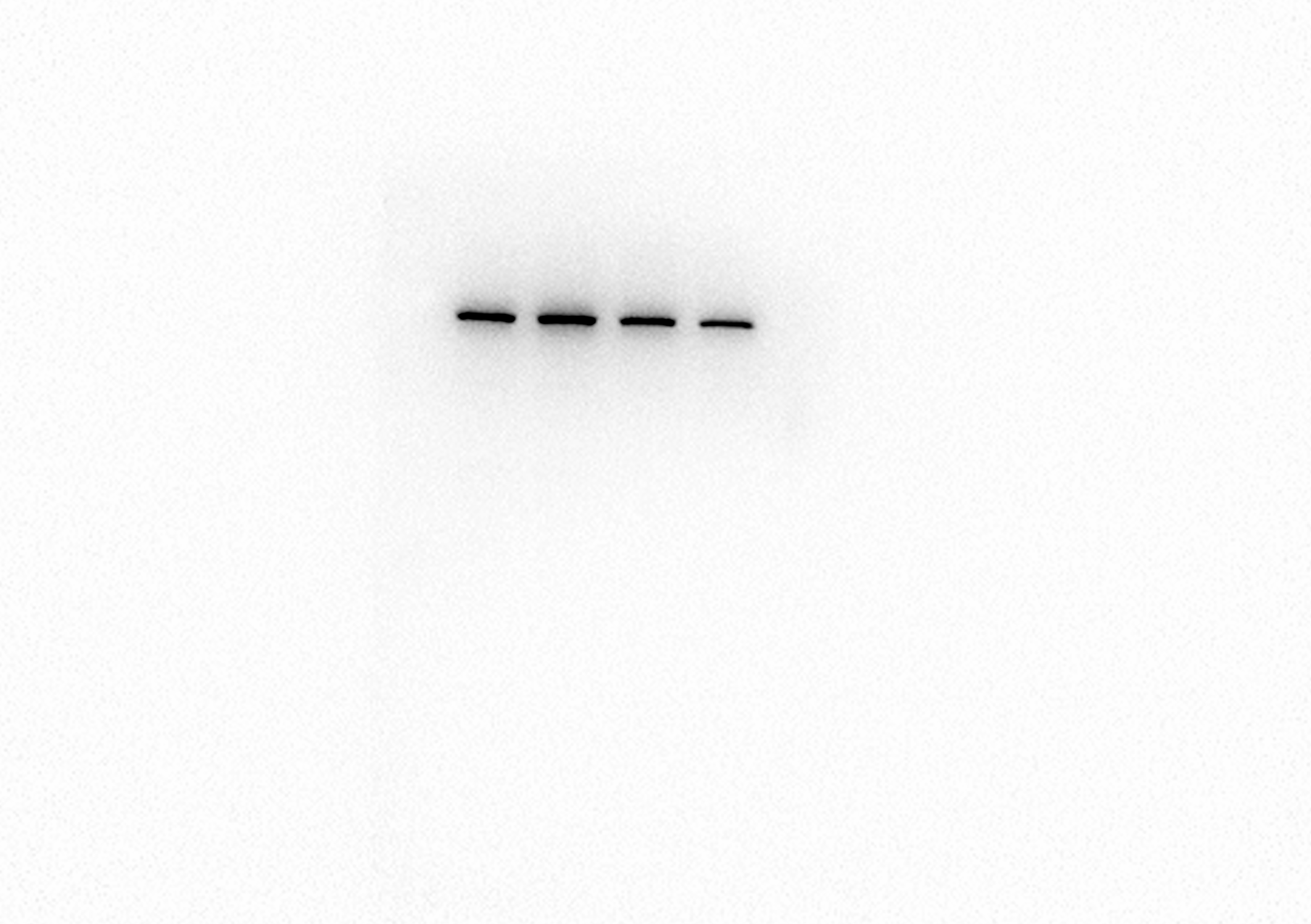

Supplement: Supplementary file 1 [file biomolecules-14-00672-s001.zip › Supplementary information S2 (original images of WB)/p-NF-kBó┘.tif]

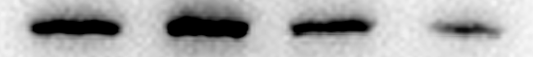

Supplement: Supplementary file 1 [file biomolecules-14-00672-s001.zip › Supplementary information S2 (original images of WB)/p-NF-kBó┌ screenshot.tif]

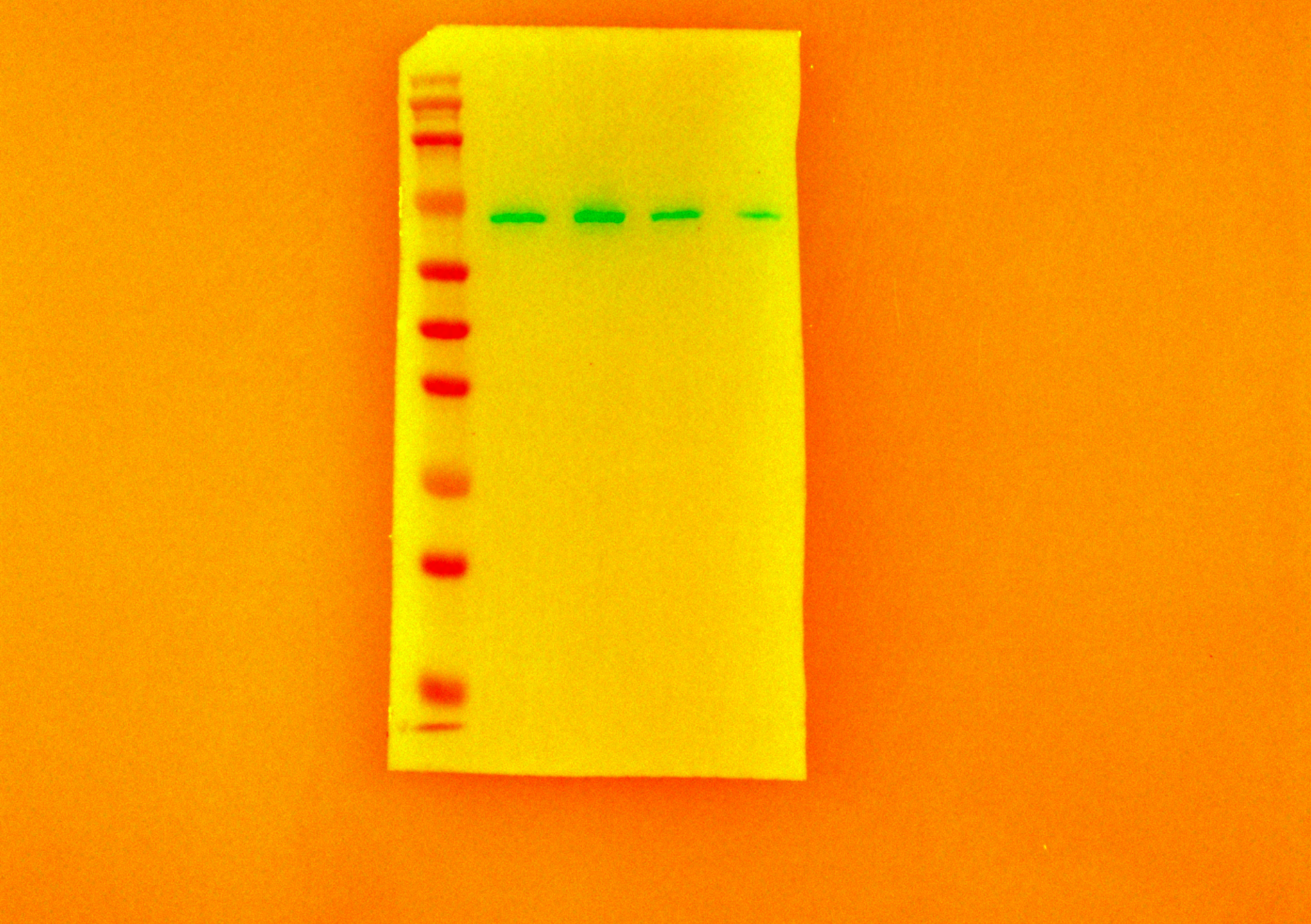

Supplement: Supplementary file 1 [file biomolecules-14-00672-s001.zip › Supplementary information S2 (original images of WB)/p-NF-kBó┌-1.tif]

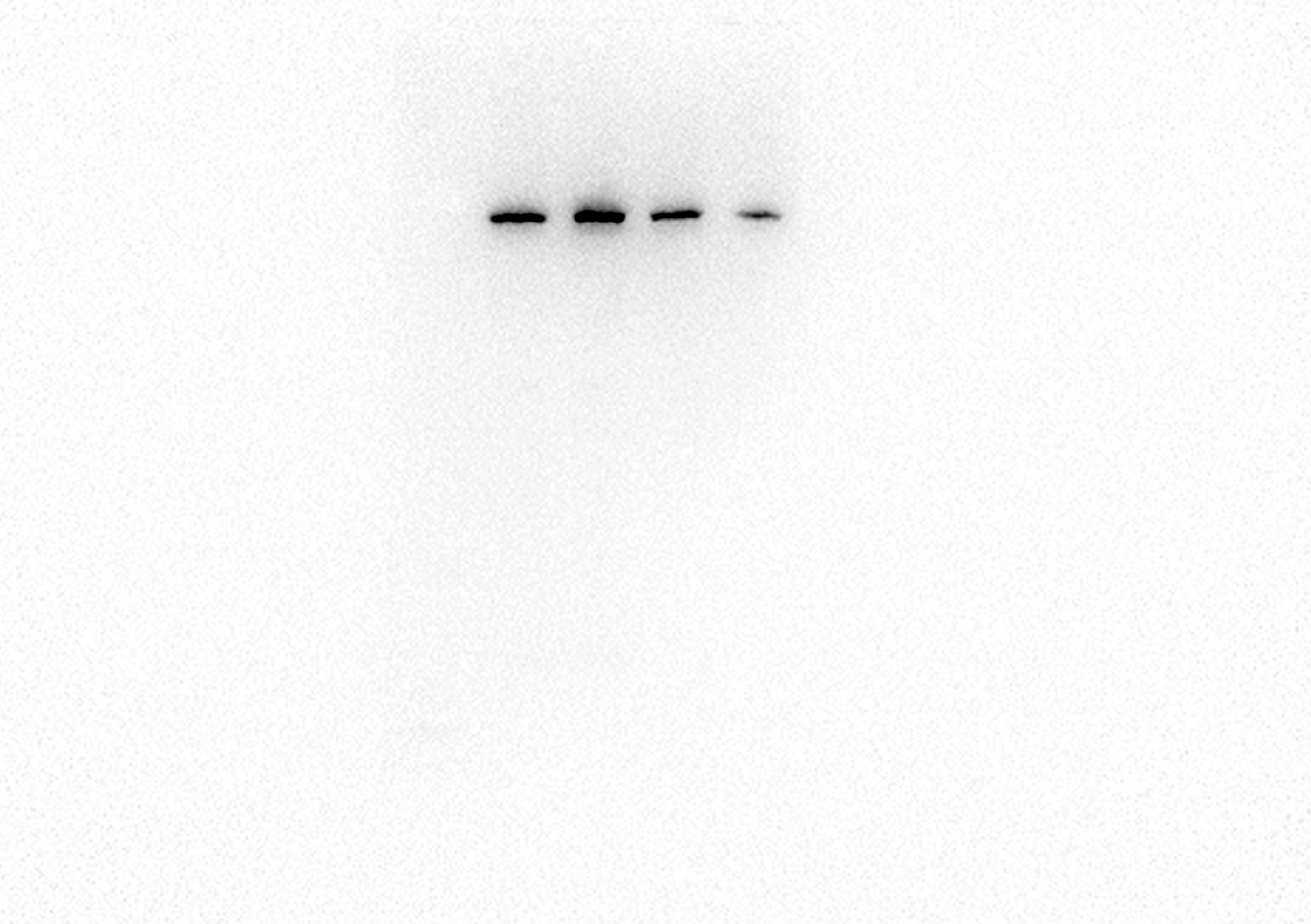

Supplement: Supplementary file 1 [file biomolecules-14-00672-s001.zip › Supplementary information S2 (original images of WB)/p-NF-kBó┌.tif]

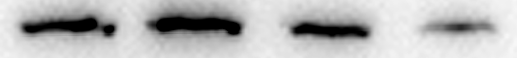

Supplement: Supplementary file 1 [file biomolecules-14-00672-s001.zip › Supplementary information S2 (original images of WB)/p-NF-kBó█ screenshot.tif]

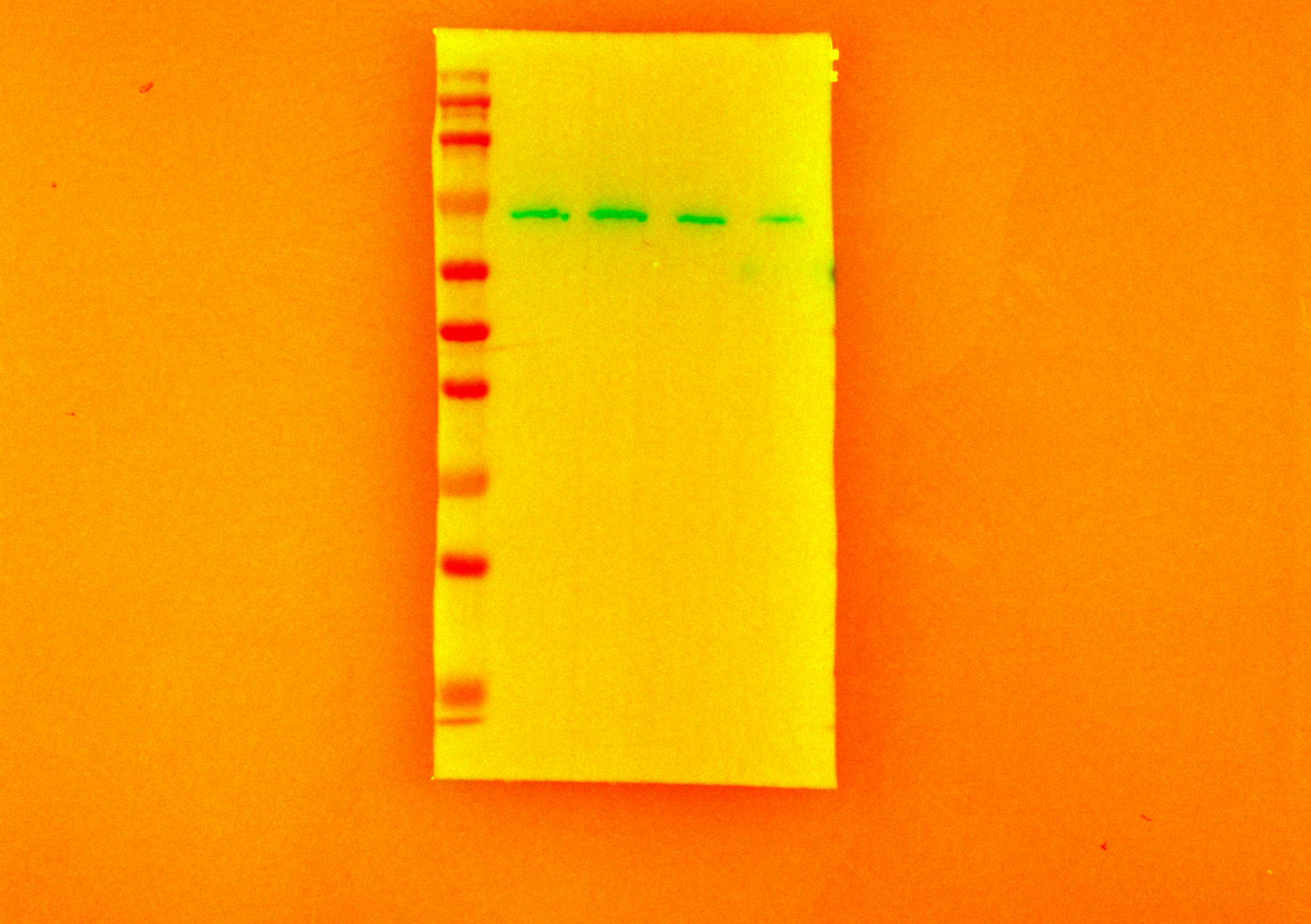

Supplement: Supplementary file 1 [file biomolecules-14-00672-s001.zip › Supplementary information S2 (original images of WB)/p-NF-kBó█-1.tif]

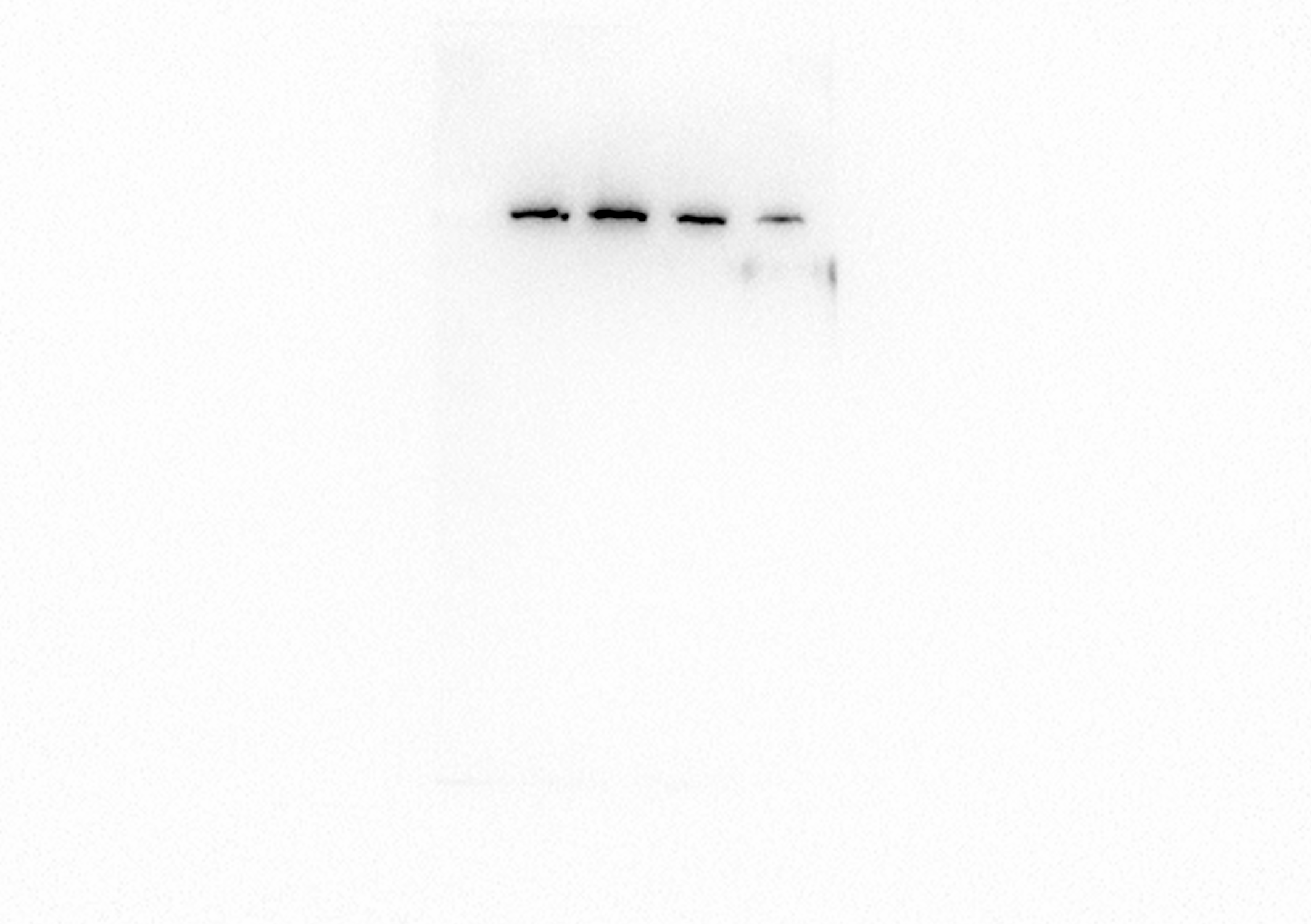

Supplement: Supplementary file 1 [file biomolecules-14-00672-s001.zip › Supplementary information S2 (original images of WB)/p-NF-kBó█.tif]
